# Solid Electrolyte Interface in Zn-Based Battery Systems

**DOI:** 10.1007/s40820-022-00939-w

**Published:** 2022-10-19

**Authors:** Xinyu Wang, Xiaomin Li, Huiqing Fan, Longtao Ma

**Affiliations:** 1 Frontiers Science Center for Flexible Electronics, Institute of Flexible Electronics, Northwestern Polytechnical University, Xi’an, 710072 People’s Republic of China; 2 State Key Laboratory of Solidification Processing, School of Materials Science and Engineering, Northwestern Polytechnical University, Xi’an, 710072 People’s Republic of China

**Keywords:** Solid electrolyte interface, Zn-based battery, Solvated structure, Artificial SEI, In situ SEI

## Abstract

Due to its high theoretical capacity (820 mAh g^−1^), low standard electrode potential (− 0.76 V vs. SHE), excellent stability in aqueous solutions, low cost, environmental friendliness and intrinsically high safety, zinc (Zn)-based batteries have attracted much attention in developing new energy storage devices. In Zn battery system, the battery performance is significantly affected by the solid electrolyte interface (SEI), which is controlled by electrode and electrolyte, and attracts dendrite growth, electrochemical stability window range, metallic Zn anode corrosion and passivation, and electrolyte mutations. Therefore, the design of SEI is decisive for the overall performance of Zn battery systems. This paper summarizes the formation mechanism, the types and characteristics, and the characterization techniques associated with SEI. Meanwhile, we analyze the influence of SEI on battery performance, and put forward the design strategies of SEI. Finally, the future research of SEI in Zn battery system is prospected to seize the nature of SEI, improve the battery performance and promote the large-scale application.

## Highlights

- The formation mechanism of solid electrolyte interface (SEI) is analyzed based on charge distributions at the electrode/electrolyte interface and molecular orbital theory.
- The factors affecting the formation of SEI are generalized from four aspects: Zn anode, electrolyte, current density and temperature.
- The design strategies for SEI layer are proposed from regulating temperature, electric and magnetic fields.

## Graphical abstract

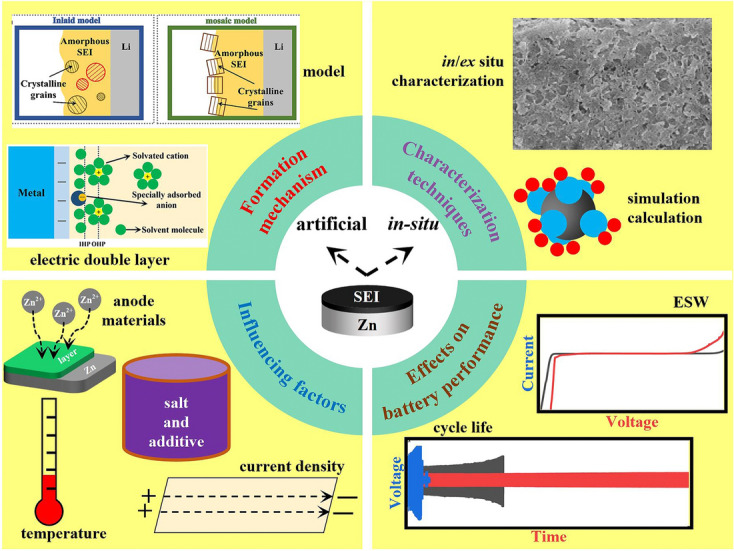

## Introduction

The development of clean new energy and the improvement of energy efficiency have become a hot topic since lithium-ion batteries (LIBs) were regarded as an efficient energy storage device in 1992. Meanwhile, LIBs account for most of the electrochemical energy market [1–6]. Although LIBs have been known for their high delivered capacity, high output voltage, high energy density and long lifespan [7–13], some negative effects including high cost, poor safety and contamination originating from limited Li sources and flammable organic electrolytes, block the future development of large-scale application. Compared with alkali metal of sodium (Na) and potassium (K), and multivalent metal of magnesium (Mg) and aluminum (Al), Zn metal has a highly theoretical gravimetric capacity of 820 mAh g^−1^ and a volumetric capacity of 5855 mAh cm^−3^ with two-electron transfer redox reaction (Table 1). Thus, it is possible to employ Zn-based batteries to replace LIBs in many fields with its actual output energy density and lifespan improved.

**Table 1:** Comparison of several metal-ions batteries including ionic radius, specific capacity and standard electrode potentials [14]. Copyright 2018, ACS Publication

Metal ion	Ionic radius (Å)	Specific gravimetric capacity (mAh g^−1^)	Specific volumetric capacity (mAh cm^−3^)	Electrode potential versus SHE (V)
Li^+^	0.76	3862	2066	− 3.04
Na^+^	1.02	1166	1129	− 2.71
K^+^	1.38	685	586	− 2.93
Mg^2+^	0.72	2205	3832	− 2.37
Al^3+^	0.535	2980	8046	− 1.66
Zn^2+^	0.74	820	5855	− 0.76

**Fig. 1:**
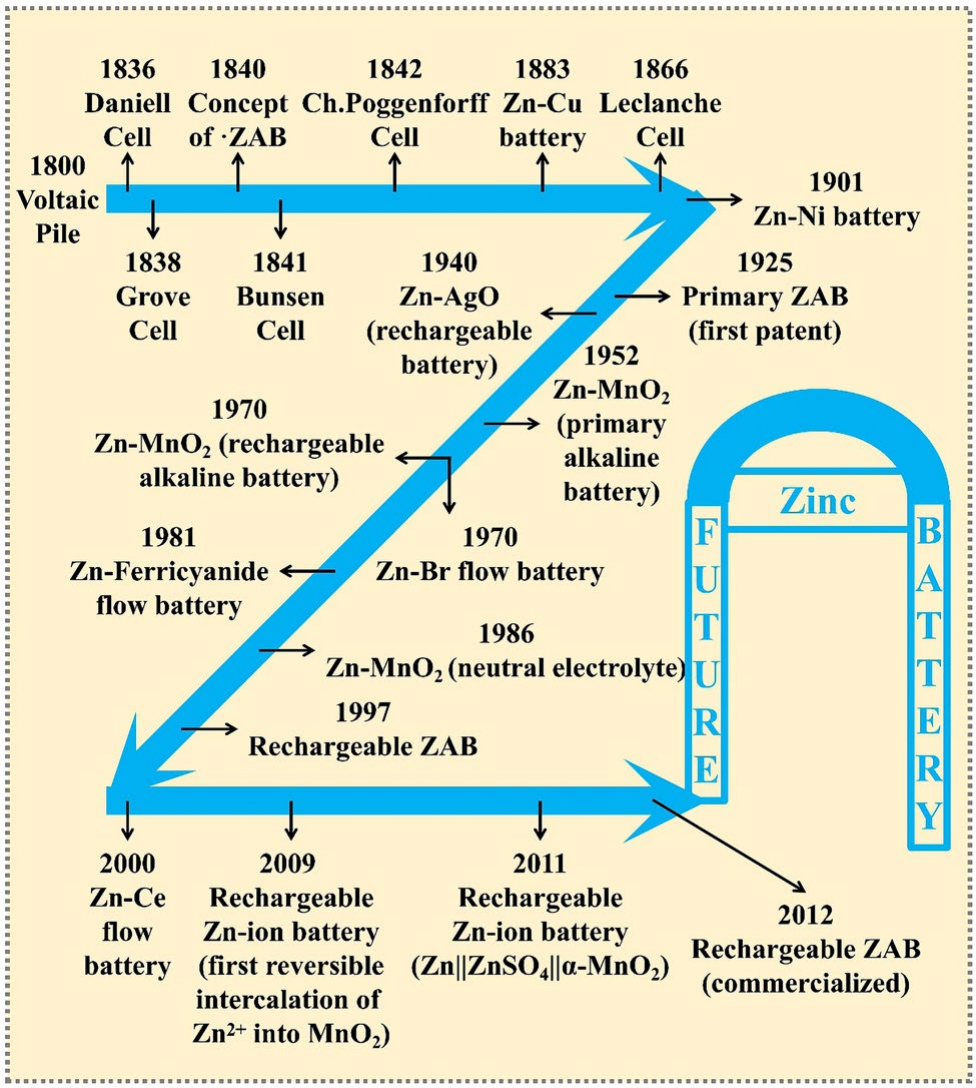
Part of the development history of Zn batteries

Throughout the research and development of Zn batteries, it has been more than 200 years since voltaic reactor appeared in 1799 [15]. The main parts of Zn batteries are depicted in Fig. 1, in which the Zn–carbon dry cell is invented by Carl Gassner in 1886 and, after that, the Henri Georges Andre Zn–Ag battery is reported in 1932, which almost dominate the battery markets. The alkaline Zn–MnO_2_ battery appears in 1952 by W.S. Herbert, and nowadays, diversified Zn ion batteries and Zn air batteries have become research hotspots. Meanwhile, neutral Zn-based batteries with plain fabrication environmental requirement and primary safety properties, also have been widely studied in the field of flexible energy storage technologies.

**Fig. 2:**
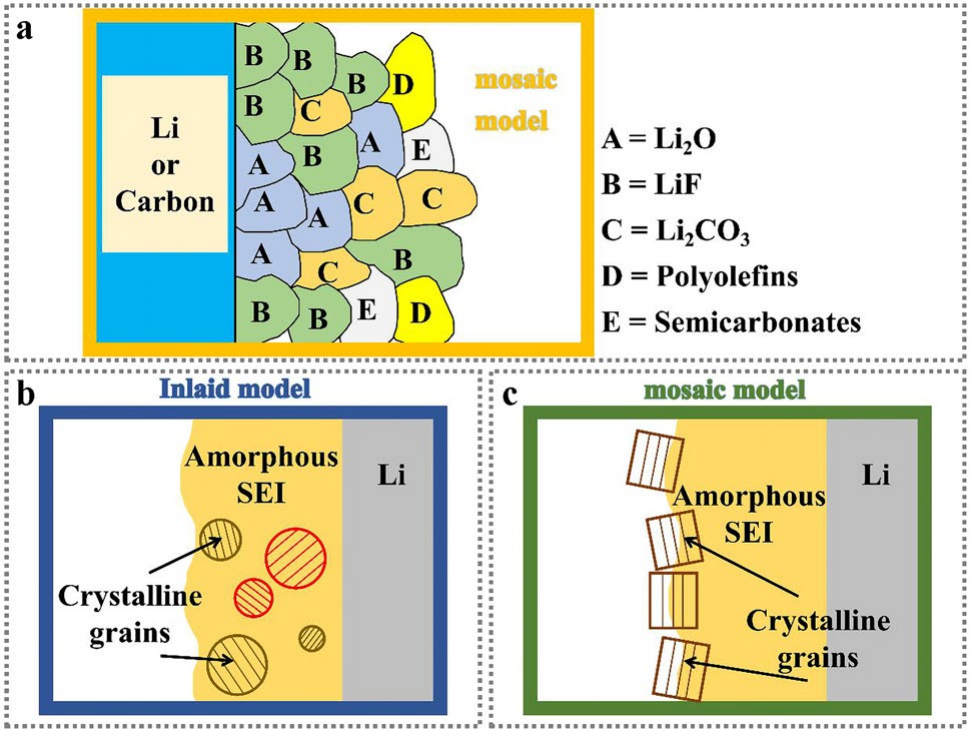
**a** Schematic diagram of traditional SEI mosaic model; **b** Schematic diagram of SEI Inlaid model [29]; Copyright 2020, Wiley–VCH. **c** Schematic diagram of multilayer SEI structure [29]. Copyright 2020, Wiley–VCH

Despite the rapid development of Zn-based batteries itself including electrodes and electrolytes, the even more serious hindrance for Zn-based batteries is the formed solid electrolyte interface (SEI) between Zn anode and electrolyte, caused by interfacial reactions. The concept of the SEI film is firstly put forward by Peled [16] in 1979, similar to LIBs with a thin layer of protective film on the surface of Li metal. After that, Aurbach in 1983 put forward the double structure of SEI film [17], in which a thin and dense layer is close to the electrode surface on one side, while a thick and porous layer is close to the side of the bulk electrolyte. To date, as depicted in Fig. 2, Mosaic model [18], Inlaid model [19] and multilayer structure model [20] have been reported for SEI layers in a variety of battery systems. When our research object is turned into Zn ion batteries, we can use similar ideas to study SEI film.

The formed SEI film with thickness from a few nanometers to dozens of nanometers refers to the interfacial reactions between electrode material and electrolyte at the interface of the two phases in initial several charge/discharge cycles. The interfacial reaction generates insoluble products with properties same to solid electrolyte, so it is noted as a SEI film [21–24]. In this process, part of Zn ions and electrolyte are consumed, which gives rise to the irreversible capacity of initial charge/discharge. The SEI film with insolubility of organic, inorganic or organic/inorganic hybrid compounds can isolate bulk electrolyte from Zn anode, preventing the solvent molecules in bulk electrolyte to be co-embedded in the deposited Zn, thus reducing the charge/discharge effect of the Zn anode. As a result, the SEI film can stabilize the Zn anode in aqueous and/or non-aqueous electrolytes and improve the service life of Zn batteries [25–28]. For an ideal SEI film, it should have the following characteristics: (1) good Zn ion conductivity and electronic insulation; (2) excellent thermal and chemical stability to reduce decomposition reaction caused by high temperature or chemical environment and to prohibit the generation of new impurities; (3) uniform and compact to physically isolate electrolyte and Zn anode direct contact and thus prevent further reduction of electrolyte solvents; (4) excellent mechanical modulus to maintain stability and deformation under stress or volume changes; (5) smaller thickness to reduce the resistance of the entire battery system.

In the Mosaic model, various inorganic and organic components are generated by the irreversible reduction decomposition of the electrolyte, and they are in the state of random distribution. This uneven distribution of components causes different ions to overcome different energy barriers in the process of transport, resulting in the difference of ion transport, macroscopic manifestations of dendrite and other phenomena. In the multilayer model, organic and inorganic components are defined as uniformly distributed, with inorganic components in low oxidation state distributed near the inner side of the electrode surface and organic components in high oxidation state distributed on the outer side. But inside the individual monolayers, the different chemical compositions are still randomly distributed, suggesting that the models are interconnected.

Using cryoelectron microscopy, the morphologies of SEI films with different structures are observed during the process of Li extraction. Lithium metal with Mosaic structure SEI extraction is not uniform, thus forming a large amount of lithium metal that loses contact with the electrode, resulting in the reduction of battery cycle efficiency. In contrast, lithium metal with multilayer structure SEI extraction evenly, the residual "dead Li" is less, so the cycle efficiency is higher. In addition, after analyzing the distribution of inorganic substances (Li_2_O, Li_2_CO_3_, etc.) nanoparticles in SEI, they found that the reasons for this phenomenon are as follows: In the Mosaic structure SEI, the density of inorganic nanoparticles is not uniform. In the area with high content of inorganic nanoparticles, the conduction speed of Li ions is fast and the speed of Li removal is fast. When the lithium metal there is consumed, the remaining metal lithium cannot maintain the path with the electrode and becomes dead Li. In the multilayer structure SEI, the density of inorganic nanoparticles is relatively uniform, and the conduction rate of Li ions is similar everywhere, so that lithium can be extracted uniformly.

In addition, the formation process of SEI film synchronously involves multiple reactions, which is relatively complex. Meanwhile, the SEI film is very sensitive to air, water vapor and carbon dioxide [30–32]. Accordingly, the analysis and characterization needs to be carried out under the condition of vacuum, which is very demanding for the detection environment [33]. Miscellaneous in situ and ex situ characterization techniques have been exploited to analyze and detect the nature and properties of SEI films, including the surface morphology, composition, thermal stability, deformation resistance, which can be related to the overall performance of the battery systems. For example, the rate capability of battery will be affected by the Zn^0^/Zn^2+^ deposition/dissolution kinetics, at which Zn^2+^ ions migrate through the SEI film [34]. Meanwhile, the Zn anode will appear some morphological and volumetric changes in charge/discharge process. On the other hand, the mechanic properties play key role in elevating battery performance. The capability of SEI film to adapt changes, determines whether it will break during electrochemical process. The fractures of SEI film enable Zn anode to directly contact with bulk electrolyte, consequently giving rise to the around-the-clock reduction of the Zn^2+^ ions to Zn^0^ at interface of electrolyte/electrode and thus continuous electrolyte depletion. The irreversibly repeated damage and formation of SEI bring about thick and non-uniform SEI film brings about capacity loss, resistance increase, metal anode corrosion, poor cyclic stability, low Coulombic efficiency and dendrite growth. Therefore, various strategies have been proposed to promote the formation and optimization of SEI film, and regulate electrode/electrolyte interface to extend the battery service life and electrochemical stability window, like employing high concentrated salt [35–37], adding organic/inorganic additives [38–40] and even artificial coating layers [41–48].

In this article, we introduced basic cognition of the SEI film, elucidated SEI formation mechanism, summarized characterization techniques and analyzed the influence factors of electrode/electrolyte on the formation and operation SEI film in Zn battery systems. Meanwhile, the influence factors of SEI films on battery performance were also discussed. After that, some strategies for constructing SEI and modifying natural SEI film were proposed to improve the Zn battery performance. Finally, the future research of SEI film in Zn battery systems was prospected.

## Formation Mechanism of SEI

**Fig. 3:**
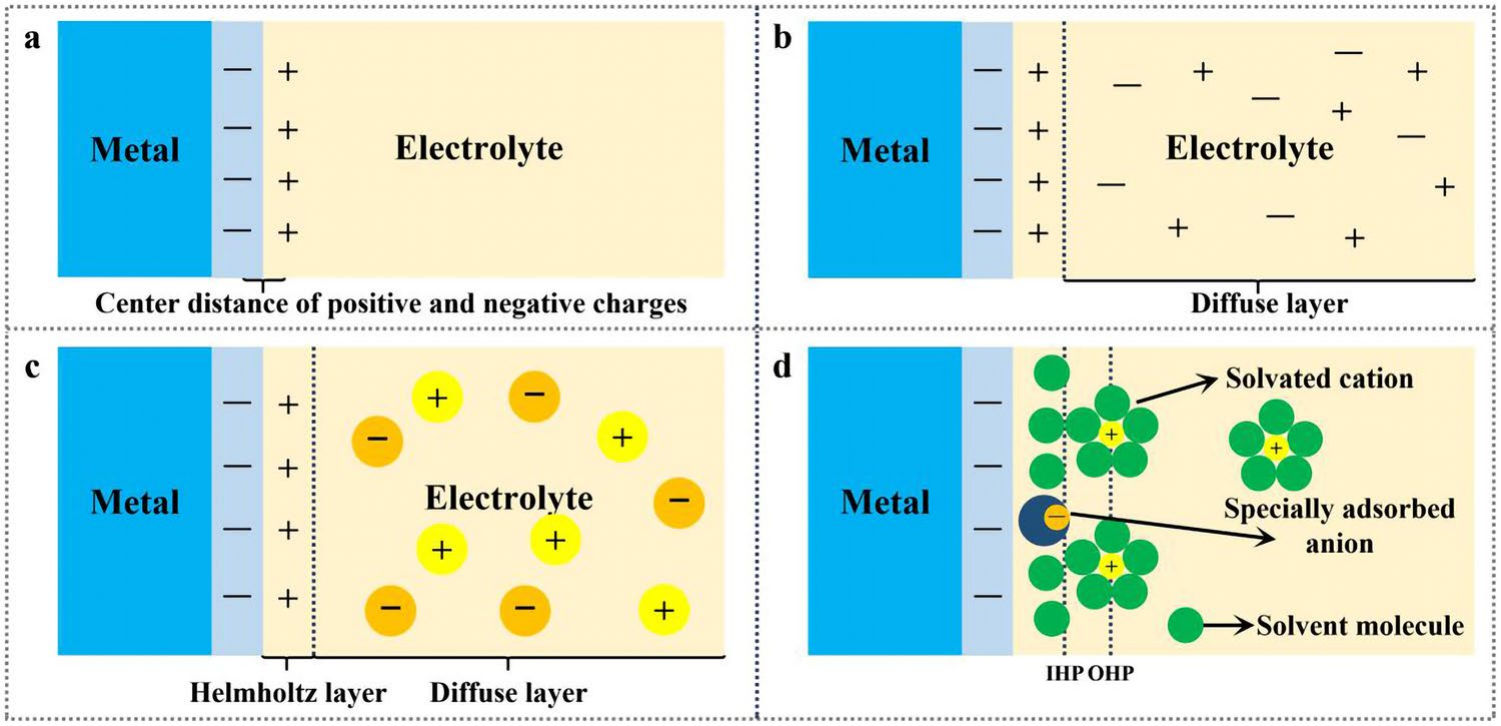
**a** Helmholtz model, the distance between positive and negative charges is a definite data; **b** Gouy–Chapman model considering diffuse layer, the distance between positive and negative charges is a variable; **c** Gouy–Chapman–Sternsilver model, ions have dimensions; **d** Electric double layer structure involving cation solvation

The model that opposite charges are equally distributed on both sides of the interface without considering the influence of relative potential and electrolyte concentration, is established based on charge distribution at the interface of electrode/electrolyte (Fig. 3a) [49]. Taking into account the influence of potential difference and electrolyte concentration, a new concept of diffusion layer is proposed on the basis of the original model (Fig. 3b) [50]. Nevertheless, there is a deviation between theory and practice in the extreme potential difference. With review that charged ions have size other than particles ignoring radius in original model, the layer structure model is defined with the dense layer as the inner Helmholtz plane and the diffusion layer is denoted as the outer Helmholtz plane (Fig. 3c) [51]. In actual electrolyte, Zn ions and anions are surrounded by solvent ions and the adsorption among particles will eventually affect the formation and properties of the SEI film (Fig. 3d) [52].

The decomposition products of solvents, solvated cations and additives are the main components of the dense layer of SEI film [53–56]. The chemical composition and electric double layer (EDL) play an important role in the structure of SEI dense layer. Some characteristic adsorption occurs in the inner Helmholtz plane and no characteristic adsorbents except solvent molecules appears in the diffusion layer. For instance, the characteristic adsorption of saccharin-derived anions in the initial electric bilayer structure facilitates the formation of water-poor bilayer structure and regulates the Zn^2+^ diffusion in deposition process, thus preventing direct contact between some water molecules and Zn metal, and prohibiting the occurrence of side reactions and dendrites growth [40]. With ZnCl_2_− dimethyl sulphoxide (DMSO) −H_2_O electrolyte as example. the preferential solvation shell of ZnCl_2_^−^ DMSO −H_2_O and the strong interaction of DMSO-H_2_O hinder the decomposition of solvated H_2_O and free water, as well as the Zn dendrite growth [57].

Before the battery is charged/discharged for the first time, and before any interphase chemical reactions occur, a double electric layer forms at the electrode/electrolyte interface due to the self-combination of solvent molecules, as well as the influence of Li ions and electrode surface potentials [58]. This double layer determines the phase interface chemistry of the cell; when the electrode surface is negatively charged, it repels anions from the inner layer, resulting in a thin and dense inorganic SEI inside. This dense layer conducts metal ions and insulates electrons. After the inner SEI layer is formed, the outer SEI layer, which is rich in organic molecules, can be penetrated into the electrolyte. In the presence of a high concentration of fluorine-rich electrolyte, fluorinated SEI is formed due to the presence of anions in the bilayer. Such real-time nanoscale observations will help design better interfaces for future batteries.

According to the electrical double layer on the basis of SEI, under the condition of Zn-based batteries have the formation of SEI, the inner layer can form inorganic SEI, might contain ZnF_2_, ZnO and inorganic elements, and form dense inorganic SEI, excellent mechanical properties, the dendrite formation have inhibition effect, also can cut off direct contact with Zn electrode, electrolyte Prevent hydrogen evolution and side reactions. And outer main associated with irreversible reduction of electrolyte decomposition, can form organic SEI, porous, flexibility good characteristics, it also showed the compatibility of SEI and electrolyte, for the subsequent Zn ion transport, and adherent inner inorganic SEI, make whole SEI smooth and effective to prevent the cracking of the SEI.

**Fig. 4:**
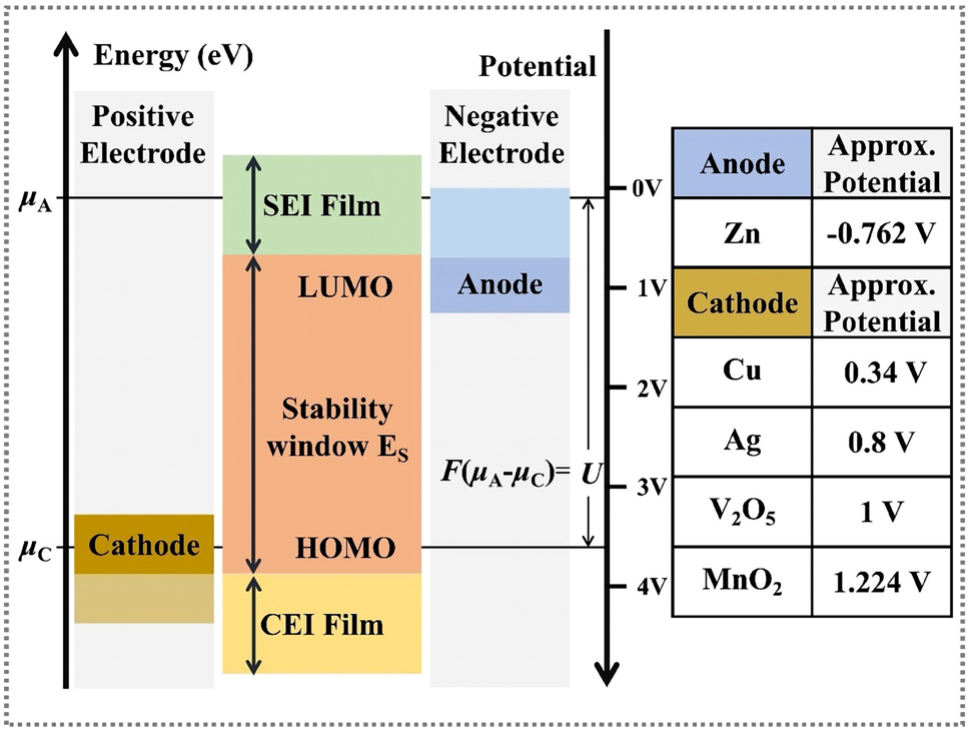
Schematic diagram of energy band of electrolyte

Furthermore, the formation mechanism of SEI films can be briefly explained by molecular orbital theory. As the energy band diagram of electrolyte shown in Fig. 4, the energy difference between the lowest unoccupied molecular orbital (LUMO) and the highest unoccupied molecular orbital (HUMO) is electrochemical stable potential window [14]. The thermodynamic stability of electrolyte solution is determined by the energy difference between Fermi level of anode and lowest unoccupied molecular orbital of electrolyte. Upon the LUMO level of the electrolyte is lower than the Fermi level of the anode, the electrolyte is thermodynamic unstable. Meanwhile, the electrolyte solution will obtain electrons from the anode and the electrolyte reduction reaction will occur.

## Characterization Techniques of SEI

Since the concept of SEI film is proposed 40 years ago, various characterization techniques have been adopted to study SEI film. Nevertheless, their formation mechanism, structure, composition and properties of SEI are still not clearly expounded. The in situ and ex situ characterization techniques are very helpful to study multitudinous internal and external properties of SEI [30, 31]. The important factors affecting the morphology, structure, composition and properties of SEI layer can be analyzed by testing the ambient temperature, current density, electrode materials and electrolyte components [32, 33]. Among them, in situ characterization technology can provide information in the process of electrochemical operation, displaying the formation process of SEI in real time and avoiding the influence of contamination by external environment.

**Fig. 5:**
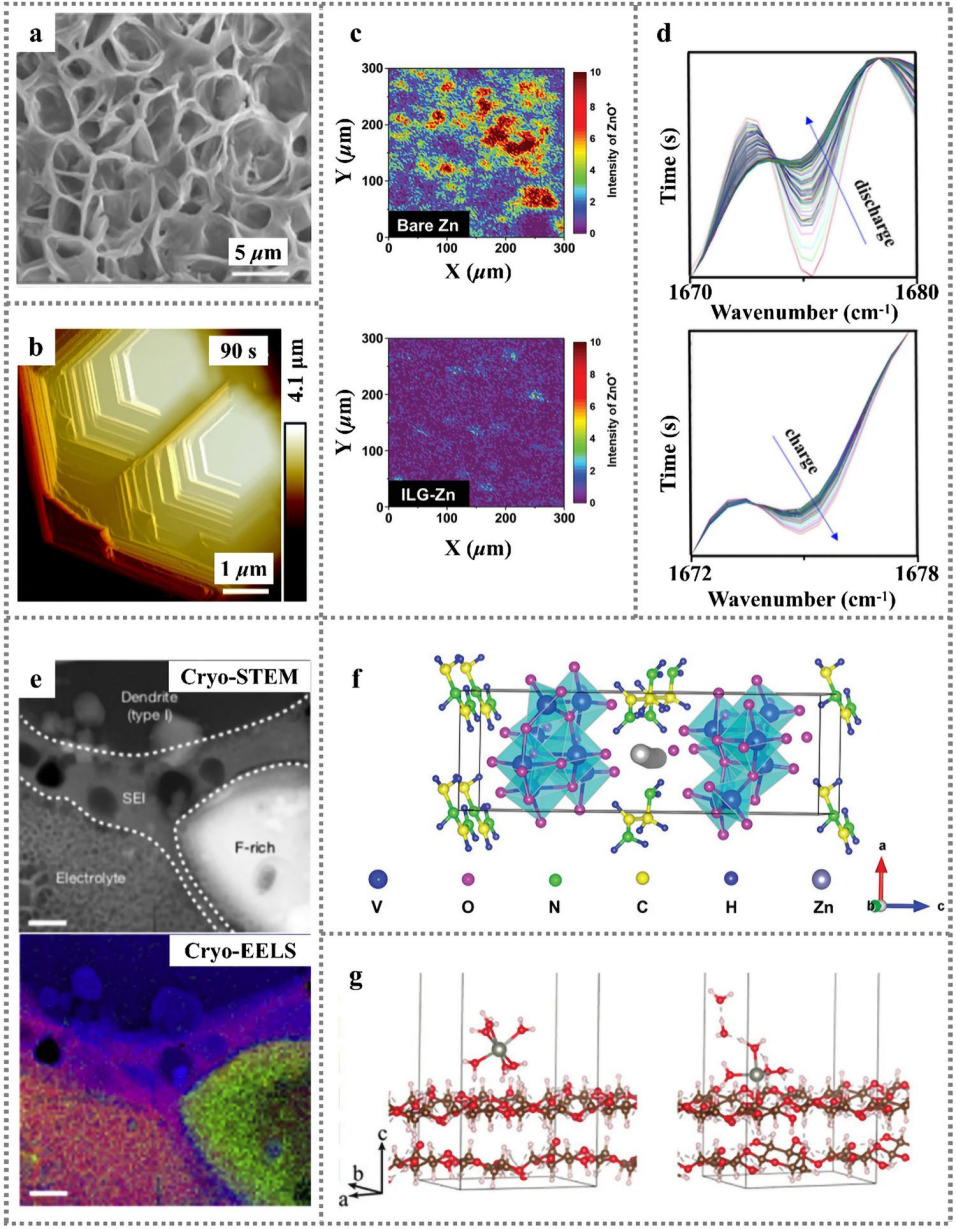
**a** SEM images of the morphologies grown at the Zn anode after plating/stripping [59]; Copyright 2019, Wiley–VCH. **b** In situ AFM images of Zn electrodeposits [61]; Copyright 2021, Royal Society of Chemistry. **c** TOF–SIMS mapping images (ZnO^+^ species) of the bare Zn (top) and ILG-Zn (bottom) [63]; Copyright 2021, Wiley–VCH. **d** 2D patterns of in situ ATR-FTIR spectra in first discharge and charge cycle [64]; Copyright 2022, Elsevier. **e** HAADF cryo-STEM imaging reveals an extended SEI layer on the dendrite, and element distribution in dendrite [66]; Copyright 2018, Elsevier. **f** Schematic illustration of Zn-ion diffusion pathway in EDA-VO crystal [69]; Copyright 2022, Wiley–VCH. **g** MD simulation of the desolvation process at the CNW surface [70]. Copyright 2021, Royal Society of Chemistry

Scanning emission microscopy (SEM), transmission emission microscopy (TEM), atomic force microscopy (AFM) and other electron microscope analysis techniques can be used to characterize the surface morphology and microstructures of SEI films. For instance, the hybrid solvent of triethyl phosphate (TEP)-water (TEP-H_2_O) enables highly porous and dendrite-free Zn coatings with stable Zn deposition/dissolution cycles. From the SEM image, the uniform and apatite-like sediments with porous interconnection structure significantly increases the surface volume (Fig. 5a) [59]. The initial state and process of Zn anodic electrodeposition with high spatial resolution is studied by in situ TEM [60]. The dissolution of dendrites after growth starts from the root to the top of the dendrite, indicating the existence of concentration gradient between the electrode surface and the surrounding environment before the dendrite formation. The allometric growth behavior of electrodeposited Zn at initial nucleation and growth/dissolution stages is observed by in situ AFM, revealing horizontal radial growth and top-down dissolution [61]. Meanwhile, by in situ surveying the morphology change of Zn at the initial stage of electroplating (Fig. 5b), it concluded that the Zn deposition could not get rid of the influence of the substrate in a short time [62].

X-ray photoelectron spectroscopy (XPS), time-of-flight secondary ion mass spectrometry (TOF–SIMS), Fourier-transform infrared spectroscopy (FT-IR), Raman spectra and other spectral analysis techniques can be used to analyze the composition of SEI films. The SEI composition on Zn anode is analyzed by XPS coupled with Ar^+^ sputtering depth profile [57]. With ZnCl_2_–DMSO–H_2_O electrolyte utilized, after a few charge/discharge cycles, XPS analysis confirms that Zn_12_(SO_4_)_3_Cl_3_(OH)_15_·5H_2_O−ZnSO_3_–ZnS SEI is formed on the Zn anode, which conducts Zn^2+^ and obstructs water molecules from bulk electrolyte. The contribution of DMSO to SEI formation is closely related to the solvation structure of Zn^2+^ ions. The DMSO replaces the H_2_O of the Zn^2+^ solvent sheath. At the same times, the preferential solvation of DMSO with Zn^2+^ and strong H_2_O-DMSO interaction inhibit the decomposition of solvated H_2_O. Apart from organic additives, the hydrophobic ionic liquid (IL) solvent Zn salt and sulfhydryl polymer flexible skeleton incorporated in IL-based thin gel promote the formation of fewer ZnO^+^ ions on LG-Zn surface, resulting in the distribution of ZnO confirmed by TOF–SIMS. It indicates that IL thin gel prevents the damage of water molecules to the Zn anode (Fig. 5c) [63]. Meanwhile, the in situ SIMS can reveal the chemical composition of plane atomic layers and can be used to identify the molecular structure of organic components. The 1,4,5,8-naphthalene diimide (NI) is investigated via ex situ Raman measurements in situ ATR-FTIR(Fig. 5d), suggesting that the evolution of the C=O group in RNI-PTFE electrode is more reversible than that in CNI-PVDE electrode [64].

Thermogravimetric analysis (TGA), differential scanning calorimetry (DSC), accelerating rate calorimeter (ARC), temperature programmed desorption (TPD) and other thermoanalysis techniques can be used to detect the thermal stability of SEI films. DSC and TGA in combination with gas analysis are used to determine the main decomposition processes of graphite-lithium-nickel-manganese-cobalt oxide (NMC 111) batteries. Meanwhile, a reaction kinetics model for the thermal runaway model is established to study the thermal stability of batteries and the motion behavior of atoms and molecules [65].

In addition, cryogenic electron microscopy (cryo-EM) and other techniques have also been applied to the characterization of SEI films. Cryo-EM provides a means for preserving the original state and imaging of battery materials at the nanometer/atomic scale, resolving individual metal atoms and their interface with SEI at the atomic scale [66], as seen in Fig. 5e. Li et al. propose a simple method, that is, detecting the fracture and re-formation of bonds when solvent molecules decompose to preserve the atomic resolution image of sensitive battery materials, which reveals the detailed nanostructure of SEI film [67]. In this method, Li metal is deposited on a copper grid under standard cell conditions and the grid is then cleaned with an electrolyte. After that, the sample is immediately frozen in liquid nitrogen at ultra-low temperature to preserve its structure and other electrochemical information. Noted that in the process, the Li^+^ ions do not react with liquid nitrogen and ice.

In addition to the experimental characterization techniques, a variety of theoretical simulations have been proposed including molecular dynamics (MD) simulation, density functional theory (DFT), and COMSOL Multiphysics software etc. Through the simulation, we can have convenient and intuitive cognition of each module in the electrochemical process, which can not only save the experimental cost, but also provide information guidance for the specific experimental process. For example, the mechanism of ZnNi promoting uniform nucleation is studied by DFT calculation, in which the ZnNi exhibits stronger binding ability than bare Zn and Zn nuclei are preferred to form around ZnNi [68]. In addition, a first-principles method based on DFT was used to study the electron properties of ethylenediamine-vanadium oxide (EDA-VO) hybrid cathode. The density of states (DOS) reflects its electronic structure, as is shown in Fig. 5f. The high operating voltage and high capacity of EDA-Vo cathode is due to its unique electronic structure and reversible adsorption capacity of Zn ions. The electron states near the Fermi level, which consist of V-3*d*, O-2*p* C-2*p* and N-2*p* orbitals. It shows obvious *p–d* hybridization, indicating that the electrons inserted by Zn ions during EDA-VO discharge are well accommodated by the N, V and O sites [69].

With the deepening of the research on SEI, the characterization technology has also made great progress [71]. However, the results obtained from the detection will be greatly affected by the sample preparation. For the preparation of artificial SEI, such as coating a protective layer on the surface of the substrate, it is difficult to control the coating thickness and achieve a flat surface when the blade is used for scraping and coating. If the rotary coating method is used, the thickness of the coating can be controlled, but due to the action of centrifugal force, it cannot ensure that the composition is in a uniform state everywhere; if the electrospinning technology is used, it can only show the uniformity of the coating on a macroscopic level, and the combination of the protective layer and the substrate is not tight. For the preparation of the slurry, the composition should be able to bind tightly to the zinc sheet, can provide a uniform site for the nucleation and growth of zinc, but also can be stable in the electrolyte.

There are also many factors to consider when characterizing SEI generated in situ. When it leaves the original liquid electrolyte, the original stable state is broken, and the composition and thickness will change irreversibly.

Characterization techniques such as XPS, infrared spectroscopy, nuclear magnetic resonance spectroscopy, secondary ion mass spectrometry and cryo-electron microscopy are mainly used to characterize SEI in an ex situ state. The commonly used sample treatment method is to use solvent to wash SEI, but this washing method is easy to change the microscopic morphology. The soaking method is easy to dissolve some components. Whether rinsing or soaking, it is easy to leave some salt components on the surface, which will affect the detection results.

For the in situ SEI characterization, it is necessary to maintain strict conditions such as vacuum sealing, while some components in SEI will cause damage to the detection equipment, which is expensive, and some existing instruments are not suitable for samples containing liquid components. In addition, under the condition of variable temperature, the characterization of sample morphology will affect the accuracy of the equipment, and the results will also have a great impact. Therefore, new in situ characterization techniques should be oriented towards low-cost, simple, fast and efficient methods, and further research and design are needed.

Physical and chemical properties of SEI are a key factor affecting metal growth behavior [72]. Although there have been a large number of studies on mechanical, kinetic and chemical properties of SEI in the past, there is still no in-depth understanding of various physical and chemical properties of SEI and the relationship between various properties and metal growth behavior. At the same time, the relationship between the single nature of SEI and the growth behavior of metal is not clear.

The classical MD simulations are utilized to study a water-in-salt (WIS) electrolyte of Zn bis (trifluoroethane sulfonyl) imide (Zn(TFSI)_2_) + lithium trifluoroethane sulfonyl imide (LiTFSI), in which Zn^2+^ ions are mainly solvated by six water molecules in the first solvent shell, while TFSI^−^ anions are completely excluded in high concentration WIS electrolyte [73]. Zhang et al. construct a bifunctional cellulose nanowhisker-graphene (CNG) film to solve the problem of Zn dendrite and corrosion. MD simulation of the CNG desolvation process at the cellulose-nanowhisker (CNW) surface is shown in Fig. 5g. As a desolvation layer, CNG film prevents the collision between water molecules and Zn anode, and delays the water-induced corrosion reaction. This CNG layer with negative surface charges can simultaneously generate a deionization shock by spreading cations but screening anions to obtain redirected Zn deposition parallel to the (0002) Zn plane. The flexible toughened CNG film can withstand strong tensile force (8.54 N) and puncture force (0.10 N), and adapt well to the fluctuation of Zn anode surface during electroplating/stripping process [70].

## Influencing Factors of SEI Formation

**Fig. 6:**
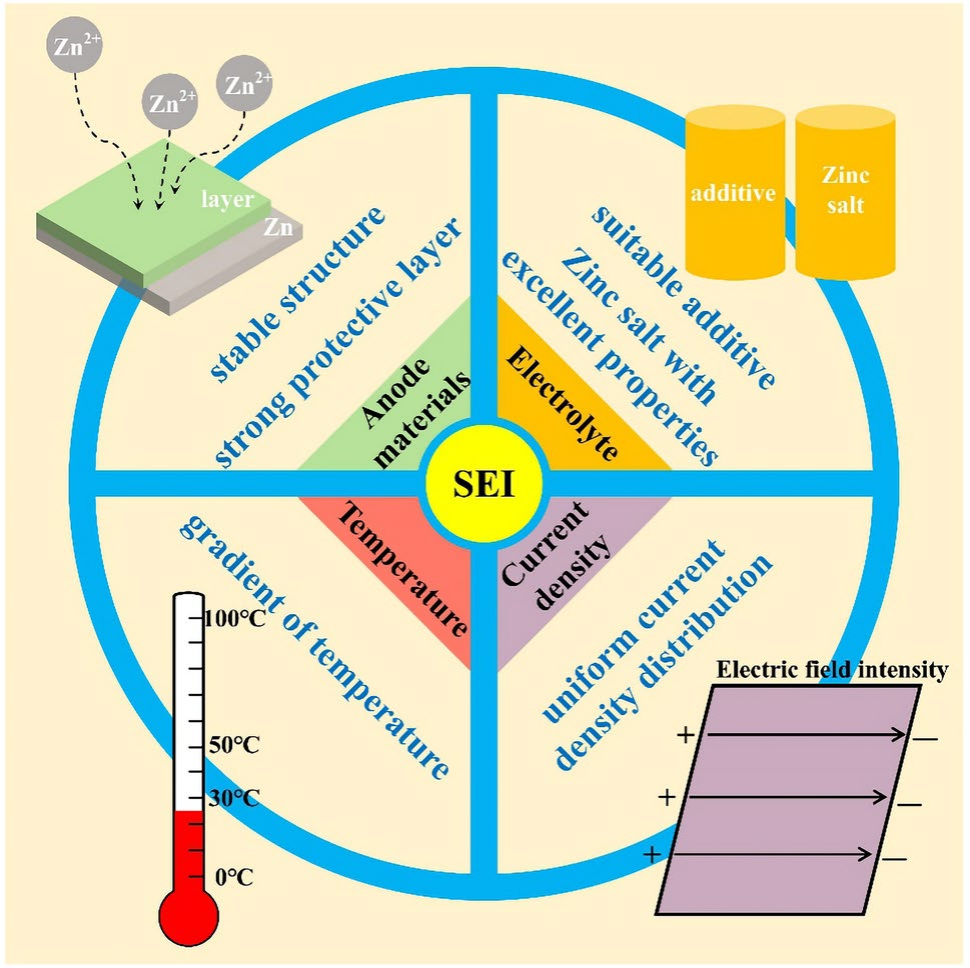
Schematic diagram of factors influencing SEI formation

The formation of SEI is generally affected by the anode material and electrolyte in direct contact with it, as well as the temperature conditions and current density in the external environment (Fig. 6).

### Anode Materials

**Fig. 7:**
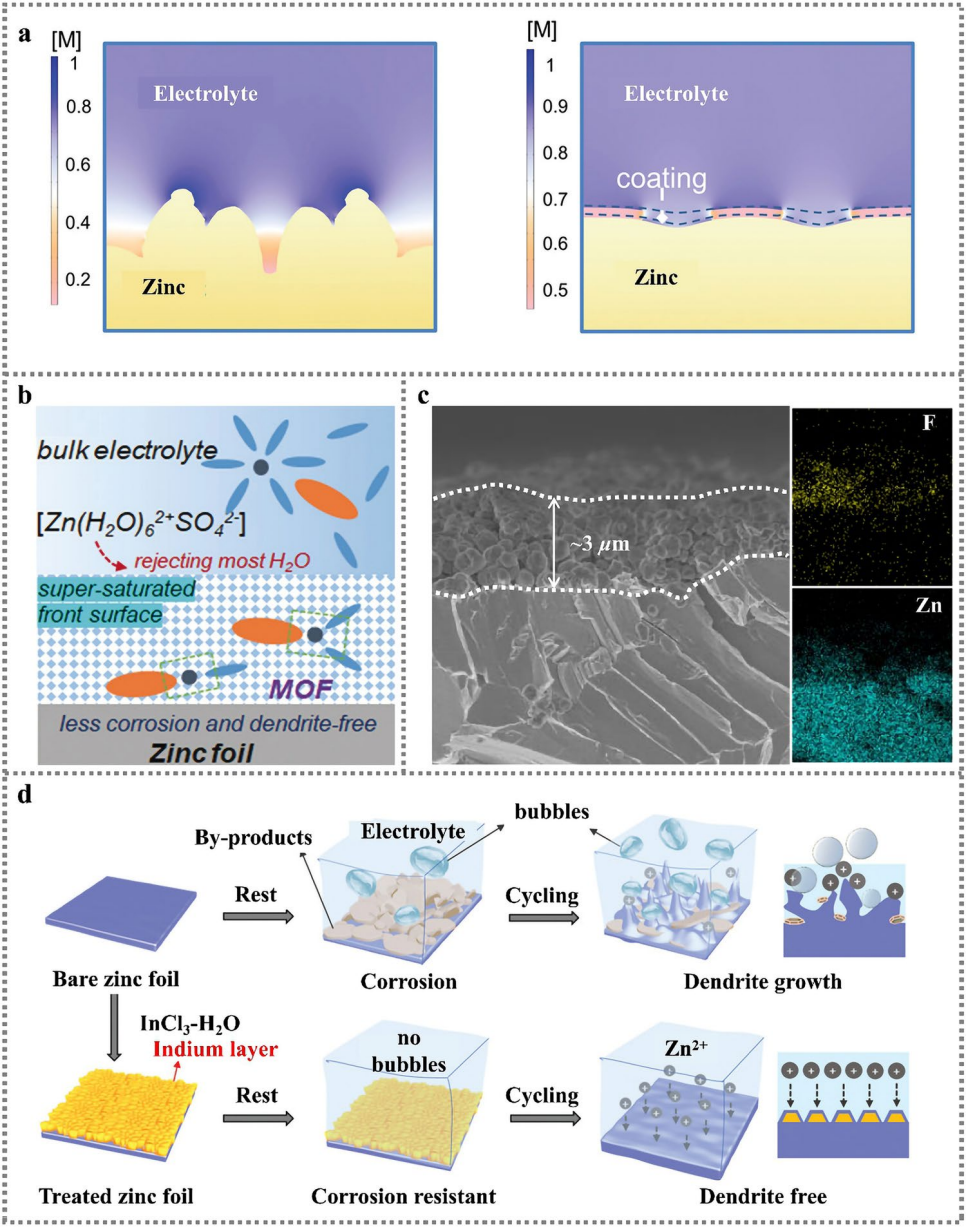
**a** The electrode surface before and after adding PDMS/TiO_2-*x*_ coating about distribution of Zn-ion concentration [75]; Copyright 2022, Wiley–VCH. **b** Function mechanism of the MOF coating layer to reject H_2_O and construct a super-saturated front surface [76]; Copyright 2020, Wiley–VCH. **c** Cross-sectional SEM image of the Zn soaked in the electrolyte and the corresponding EDX maps. Scale bar, 2 μm [77]; Copyright 2021, Springer Nature. **d** Schematics of the behavior of bare Zn and Zn|In anodes in an aqueous ZnSO_4_ electrolyte [78]. Copyright 2020, Wiley–VCH

The structural characteristics and stability of SEI are closely related to deposition/dissolution behaviors of Zn anode. As the existence of deep-seated dendrite growth, corrosion and passivation problems in Zn anode, SEI films are always non-uniform. Meanwhile, the dendrite tip can easily pierce SEI film, resulting in continuous consumption of electrolyte, as well as continuous deposition/growth at the fracture sites. The irreversible Zn deposition will give rise to the formation of “death Zn”. An option-based in situ 3D microscope is developed to observe the 3D morphology of Zn electrode in electrochemical process [74]. It is found that the electrode morphology determines the local ion concentration distribution and local current density, and further affects the Zn plating/stripping rate. Various strategies of the structure design and interface protection of Zn anodes are proposed for inhibiting side reactions and stabilizing protective layers. For example, a PDMS/TiO_2-x_ coating is utilized to adapt volume changes [75]. Meanwhile, the oxygen-rich vacancy TiO_2-x_ induces rapid and uniform Zn^2+^ transfer, which enables the assembled symmetric Zn||Zn cell to achieve a Coulombic efficiency of 99.6% after 700 cycles at a current density of 10 mA cm^−2^. The polyamide coating layer is constructed to separate the anode from bulk electrolyte solution to improve the nucleation barrier and regulate the deposition behavior of Zn (Fig. 7a). Compared with bare Zn, the operating life of Zn with polyamide coated extends to 8000 h, with 10 mAh cm^−2^ Zn cycled (10 mA cm^−2^ for 1 h, 85% depth of discharge) [47]. In addition, metal organic framework (MOF) material with unique channel structure can be used as coating layers to modify Zn anode *through* tuning specific chemical composition and porous structure (Fig. 7b). The ZIF-7 channel can prevent large solvated Zn ion complexes and supersaturated inner layer electrolyte in front of MOF channel under the action of electric field is achieved to drive uniform metal deposition [76]. An aqueous organic electrolyte composed of ZnBF_4_ and glycol solvent can form a stable layer of ZnF_2_ SEI on the surface of Zn anode (Fig. 7c), which is beneficial to inhibit the dendrite, corrosion, hydrogen evolution reaction (HER) and other side reactions. The electrolyte also demonstrates excellent non-flammability, overcoming the flammability disadvantage of organic electrolytes. The low freezing point allows it to operate normally from -30 to 40 ℃ without affecting battery performance [77]. Indium (In) has good chemical passivation property and can be used as corrosion inhibitor to protect Zn anode (Fig. 7d). A dual-functions metal In layer acts as corrosion inhibitor and nucleating agent can perfectly solve the issues of anodic corrosion and dendrite growth. The Zn|In electrode shows 54 mV ultra-low voltage hysteresis and withstands 1500 h of plating/stripping cycle [78]. The 3D nanoporous ZnO structure coating can accelerate the ions transfer and deposition kinetics of Zn^2+^ through electrostatic attraction of Zn^2+^, instead of hydrating Zn^2+^ in an electric bilayer [79]. Li et al. report a new additive: *N*,*N*-dimethylformamidium trifluoromethanesulfonate (DOTf) into ZnSO_4_ solution, in which the initial water-assisted dissociation of DOTf into triflic superacid creates a robust nanostructured SEI which excludes water and enables dense Zn deposition. Meanwhile, it effectively solves the problem of Zn dendrites and the formation of layered double hydroxide (LDH) in water system [80]. An artificial SEI with rich electron-donating functional group (cyano group) is reported for the formation the rich-cyano SEI (rc-SEI), which can improve the desolvation kinetics by formation interaction force between cyano group and water sheath of hydrated ions [81].

### Electrolytes

The components and properties of SEI film are highly similar to electrolyte, because it is formed by the reducing and decomposing electrolytes. Meanwhile, the dissolved salts and additives in electrolytes also affect the formation of SEI films.

#### Salts

Optimizing electrolyte is a more efficient and effective method to improve the battery performance, in which a large amount of inorganic/organic Zn salts is dissolved in solvent to obtain ion transport path and construct stable SEI membranes. The common inorganic Zn salts are Zn(ClO_4_)_2_, Zn(NO_3_)_2_, ZnSO_4_, ZnF_2_, ZnCl_2_, Zn(BF_4_)_2_, etc., while the organic Zn salts are Zn(CF_3_SO_3_)_2_, Zn(TFSI)_2_, etc.

Under the condition of high voltage, Cl^–^ and NO_3_^–^ in Zn salt solution are always unstable, giving rise to unsatisfactory cyclic stability. Although the solubility of ZnCl_2_ is high, the side product of ZnCl(OH)_5_·*x*H_2_O is produced and it cannot completely prohibits side reactions of HER [82]. The Zn(ClO_4_)_2_ salts-based electrolyte shows small overpotential for Zn stripping/plating, and stably cycles over 3500 h. The excellent performance is guaranteed by the controlled reduction of ClO_4_^–^ on Zn to form a Cl^–^ containing layer [83]. Although both of ZnF_2_ and ZnCl_2_ are halogen Zn salts, they show different characteristics. The ZnF_2_-based SEI layer with fewer by-products shows huge potential for stable Zn abode, but its low solubility in water prevents it from being used as the main metal Zn salt for Zn ion transport, only as an additive [84]. The ZnSO_4_ salt-based aqueous solution is most common electrolyte for AZIB, due to its advantages of low cost, good compatibility and good electrochemical stability. It enables Zn anode to exhibit rapid dissolution/deposition kinetics, weak corrosion and low dendrite growth. Meanwhile, the reversible formation/decomposition of Zn_4_(OH)_6_SO_4_·5H_2_O enables ultra-long cyclic Zn||VO_2_ batteries with aqueous ZnSO_4_ electrolyte utilized [85].

**Fig. 8:**
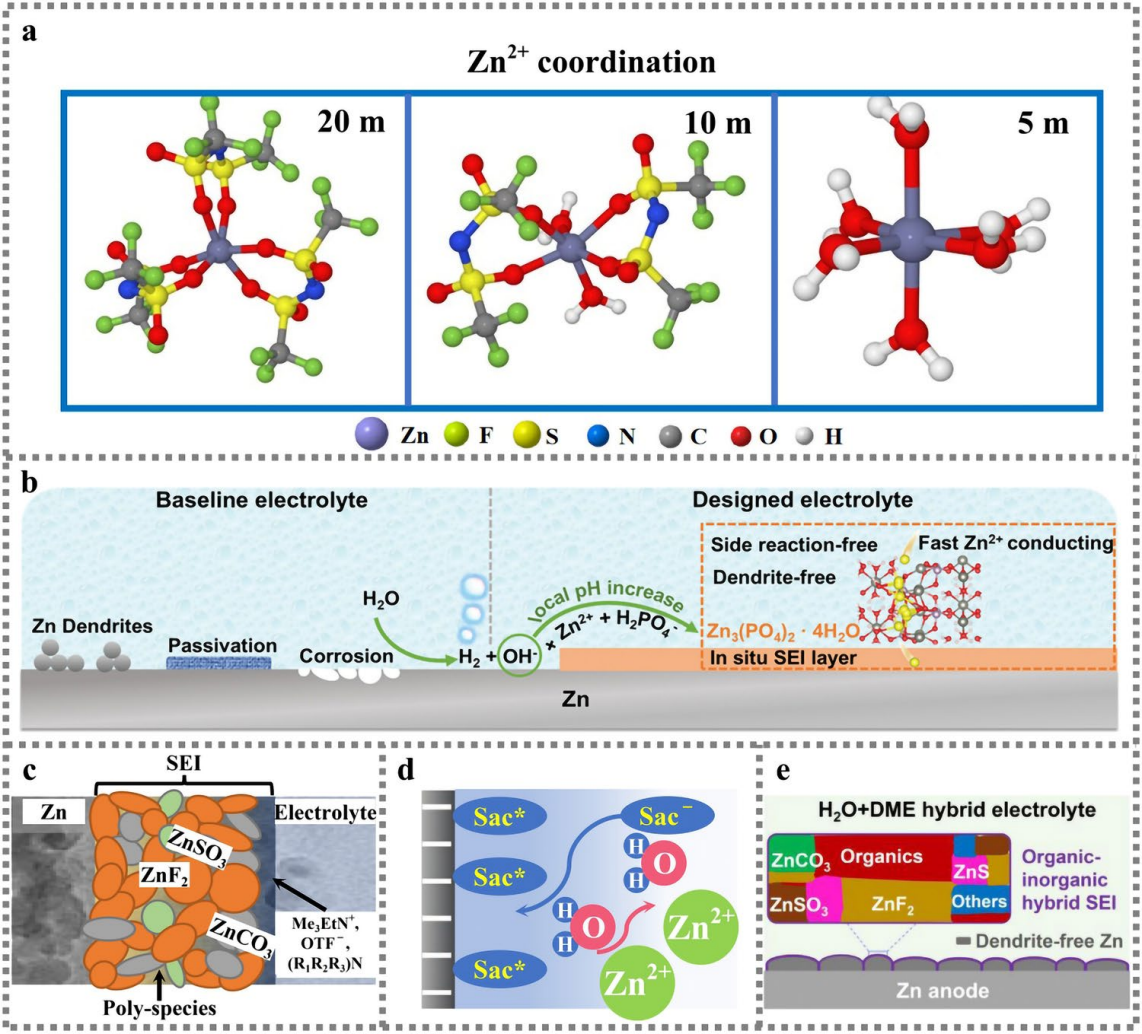
**a** Solvation structure of Zn ions in electrolytes with different concentrations [35]; Copyright 2018, Springer Nature. **b** Schematic illustration of Zn surface evolution and the SEI formation mechanism [38]; Copyright 2021, Wiley–VCH. **c** Schematic diagram of proposed Zn^2+^ conducting SEI [39]; Copyright 2021, Springer Nature. **d** The schematic descriptions of EDL structure after introducing Sac (Sac^−^ represents free Sac anions, Sac* represents the Sac anions chemically bonded with Zn surface) [40]; Copyright 2021, Wiley–VCH. **e** Schematic illustration of the interphase chemistry on Zn electrode [87]. Copyright 2022, Elsevier

Compared with the traditional inorganic Zn salt, organic Zn salt with large organic anions can provide a variety of possibilities in formation of SEI film and encourage SEI with different structures and components. The organic ingredients enhance the mechanic strength of SEI film. For instance, developed Zn(CF_3_SO_3_)_2_-H_2_O-polyethylene glycol (PEG) electrolyte possessing anion-dominated solvent structure can reduce water molecular decomposition and promote the formation of a ZnF_2_-rich SEI film, which enables symmetric Zn||Zn stably more than 9000 h with uniform Zn deposition [86]. Meanwhile, the TFSI^−^ in neutral high concentration of proppant salt of 1 m Zn(TFSI)_2_ + 20 m LiTFSI (m is mass molar concentration (mol kg^−1^), shows a unique solvent sheath structure due to the action of a large number of anions (Fig. 8a) [35]. Closed ion pair of (Zn-TFSI)^+^ is formed near Zn^2+^, significantly inhibiting the (Zn-(H_2_O)_6_)^2+^ formation. Accordingly, the prepared hybrid Zn||LiMn_2_O_4_ battery exhibits excellent cycling performance with 85% capacity retained after 4000 cycles at the CE nearly 99.9%. Although high concentrated salt endows battery excellent electrochemical performance, it has problems of high cost, high viscosity, limiting commercialization process. The electrostatic repulsion between Zn tip and anionic solvation structure inhibits the formation of dendrites. In addition, the organic/inorganic SEI interface of fluorine-rich Zn compounds are observed on the Zn anode based on the acetamide-Zn(TFSI)_2_ eutectic electrolyte [37]. The presence of anion complex Zn species significantly reduces the decomposition energy and facilitates the in situ formation of the interface, consequently achieving dendrite-free Zn deposition stability at an area capacity higher than 2.5 mAh cm^−2^. Recently, a non-flammable aqueous organic electrolyte used Zn(BF_4_)_2_ as a Zn salt and glycol as a solvent, resulting in the formation of a dense protective layer of ZnF_2_. At the same time, the non-flammable characteristics also improve the safety of using organic electrolytes, the unique bond cooperation also breaks the hydrogen bond between water molecules, realizing the stable operation at wide temperature range from − 30 to 40 ℃ [77].

#### Additive

Electrolyte additives can not only improve ionic conductivity and broaden electrochemical voltage window, but also inhibit dendrite growth and regulate solvation structure. A variety of inorganic and organic additives is utilized for Zn ion batteries to suppress Zn dendrites and prohibits Zn corrosion & passivation. The Zn(H_2_PO_4_)_2_ salt is introduced into the electrolyte to in situ form a dense and stable Zn_3_(PO_4_)_2_ SEI layer with high Zn^2+^ conductivity, which could separate Zn anode from bulk electrolyte to inhibit side reactions (Fig. 8b). Accordingly, they demonstrate a uniform Zn deposition and rapid transport kinetics [38]. Similarly, the alkyl ammonium salt additive can also promote the formation of a dense and highly Zn^2+^ conductive ZnF_2_-based SEI in dilute acidic aqueous electrolyte. The Zn(OTf)_2_ aqueous solution with trimethylethyl ammonium trifluoromethanesulfonate (Me_3_EtNOTF) additive empowers to in situ generate fluorinated hydrophobic interface phase. The SEI mainly contains ZnF_2_, ZnCO_3_, ZnSO_3_ and polyanions, which can inhibit side reactions of HER and dendrite growth, promote the Zn^2+^ transfer in charge/discharge process and extend the electrochemical stability window, thus enabling highly reversible and dendrite-free galvanization/stripping exceeding 99.9% CE (Fig. 8c) [39]. In addition, the inorganic ZnSO_4_ salt aqueous solution with saccharin (Sac) added can derive anions preferentially adsorbed on Zn metal surface to adjust the H_2_O-poor electric double layer structure (Fig. 8d) and facilitate the decomposition of Sac anions, which can modulates the Zn^2+^ diffusion kinetics and Zn^2+^ deposition behaviors [40]. With the 1,2-dimethoxy-ethane (DME) added to Zn(OTF)_2_ aqueous electrolyte, the DME molecules interrupt the original hydrogen bond network of water to form a unique solvation structure of Zn^2+^-DME-OTF^–^-H_2_O, in which DME molecules preferentially adsorbing on the Zn anode surface and consequently prevents the growth of Zn dendrites [87]. Meanwhile, through the decomposition of DME and OTF^−^, organic/inorganic hybrid ZnF_2_-ZnS SEI is in situ formed on the Zn anode, which inhibits the infiltration of Zn metal by water (Fig. 8e).

When adding some polymer or organic particles to the drainage ZIBs, the first thing to consider is the compatibility of these additives with the aqueous electrolyte, and also to ensure that they can be evenly distributed in the solution and exist stably. Many polymers or organic small molecules contain polar functional groups, which makes them zincophilic. In the process of Zn deposition, their flexibility will adapt to the change of volume and enhance the structural stability of SEI. The polar functional groups of these polymers or organic small molecules may also act on the hydrogen bond network in the original electrolyte, changing the freezing point of the electrolyte, so that the aqueous electrolyte can work at low temperatures.

Lithium metal has high activity, organic electrolytes are widely used in the LIBs, although LIBs showed excellent battery performance, but have not been effective to solve security problem, and the organic electrolyte because of its high viscosity, can produce block for the spread of the Li ion migration, resulting in a loss of conductivity. Due to its active chemical properties, lithium metal is easy to react to generate SEI. Due to the use of organic electrolyte, the composition of SEI is complex and organic matter accounts for a large proportion. Zinc can exist in water system electrolyte, but it is not easy to form SEI. When organic additives are added, the electrolyte will be irreversibly reduced and decomposed, and SEI will be formed. SEI inorganic components in zinc batteries are relatively simple. Aqueous ZIBs electrolyte show high security and the ionic conductivity, when in the water electrolyte used in LIBs, if there is no protection of SEI, water molecules are easily reacted with the lithium metal, oxide cathode, oxygen or hydrogen evolution reaction can affect the PH near electrode, lead to instability of active material and the insertion/extraction of Li ion.

Therefore, the salt, solvent and additive in electrolytes directly affect SEI layer in terms of chemical composition and structure, thus impacting the transmission efficiency of charged particles and system stability. It is necessary to explore a new electrolyte formula for the construction of ideal SEI membrane.

### Temperature

The temperature of the reaction systems also catches the reductive decomposition of electrolyte on electrode surface. When the reactions are disturbed by the external environment factors such as temperature changes, it is bound to the inborn product properties of SEI, including composition, microstructure, density, uniformity. A dense and uniform ZnS interface phase is in situ constructed on the surface of Zn at 350 ℃. The thickness of ZnS film is controlled by the treatment temperature and sulfur dosage [32]. This dense artificial ZnS layer not only inhibits Zn corrosion and hydrogen evolution by forming a physical barrier on the surface of Zn, but also prohibits dendrite growth by guiding Zn plating/stripping under artificial layer. At the appropriate temperature, the reaction energy barrier that needs to be overcome to generate SEI will be reduced, which is also conducive to the tight binding of SEI and electrode. The uniform temperature gradient also promotes the stability of SEI structure.

### Current Density

During SEI formation process, diverse ions have different transfer behaviors in term of the migration quantity and multiplier, which results in individual SEI components at different current densities. The research of Dolle concludes that the current density doesn’t have much effect on the thickness of SEI film, but it has a great influence on the composition [88]. The transport behavior of Li ions in different graphite electrodes is studied by changing the current rate to change the properties of SEI [89]. The electrode impedance is reduced, and the ion diffusion is increased with the SEI layers formed at higher current density. This is because that the adjustment of the applied current can cause the change of magnetic field. Similarly, when the Zn ions are under the action of suitable external magnetic field, it can induce the uniform deposition. A selectively polarized ferroelectric polymer ((polyvinylidene fluoride-trifluoroethylene) (P(VDF-TrFE)) as a surface protective layer on Zn anodes surface can regulate Zn ions concentrate along the surface of the film. Subsequently, Zn can evenly grow to empower the symmetrical Zn||Zn cell cycle over 2000 h at 0.2 mA cm^−2^ and 0.2 mAh cm^−2^ [90]. The homogeneous electric field induced the migration of Zn ions, alleviated the adverse effects caused by the tip effect, prevented the uneven distribution of ion concentration in the system, and well inhibited the formation of dendrites.

## Effects of SEI on Battery Performance

Although Zn ion batteries has a great application prospect in flexible wearable devices and energy storage due to the advantages of low cost, high safety and ideal specific capacity, the dendrite growth, some side reactions of Zn anode corrosion and passivation and hydrogen evolution have a non-negligible negative impact on Zn ion battery performance, including low cycle lifespan, poor rate capability, low Coulombic efficiency and narrow electrochemical stability window. Encouragingly, these issues can be meliorated by various kinds of SEI films.

Zn ion battery in the process of cyclic charge/discharge is accompanied by partial reduction of electrolyte for the formation of SEI film. Nevertheless, the reduction product of aqueous solution contains gas, which leads to mechanical cracking or shedding of SEI films. In this case, the growth of dendrite pierces separators and the gas swells the battery to bring about short circuit problems and battery bursts, which is the long deep-seated challenge. In order to solve above problems, the Zn metal is covered by artificial SEI layers to isolate Zn anode from bulk electrolyte. These artificial SEI layers can usually guide the uniform Zn ions deposition/stripping to achieve the dual function of "protection and guidance", but there are also some problems such as tedious preparation process and poor compatibility between the anode and the protective layer. Another strategy is to optimize the electrolyte system to in situ form SEI film. A small part of the electrolyte is reduced for SEI formation, which is better than artificial SEI. Meanwhile, it also improves the coordination structure of Zn ions in the electrolyte, enhancing the ion migration ability and greatly increasing the ionic conductivity. Nevertheless, if Zn dendrites pierce the in situ SEI film, it will continue to form a new SEI film at the crack and become thicker and thicker, which will cause a sharp increase in internal resistance, the consumption of vast electrolyte and sharply capacity attenuates.

### Cycle Life and Coulombic Efficiency

**Fig. 9:**
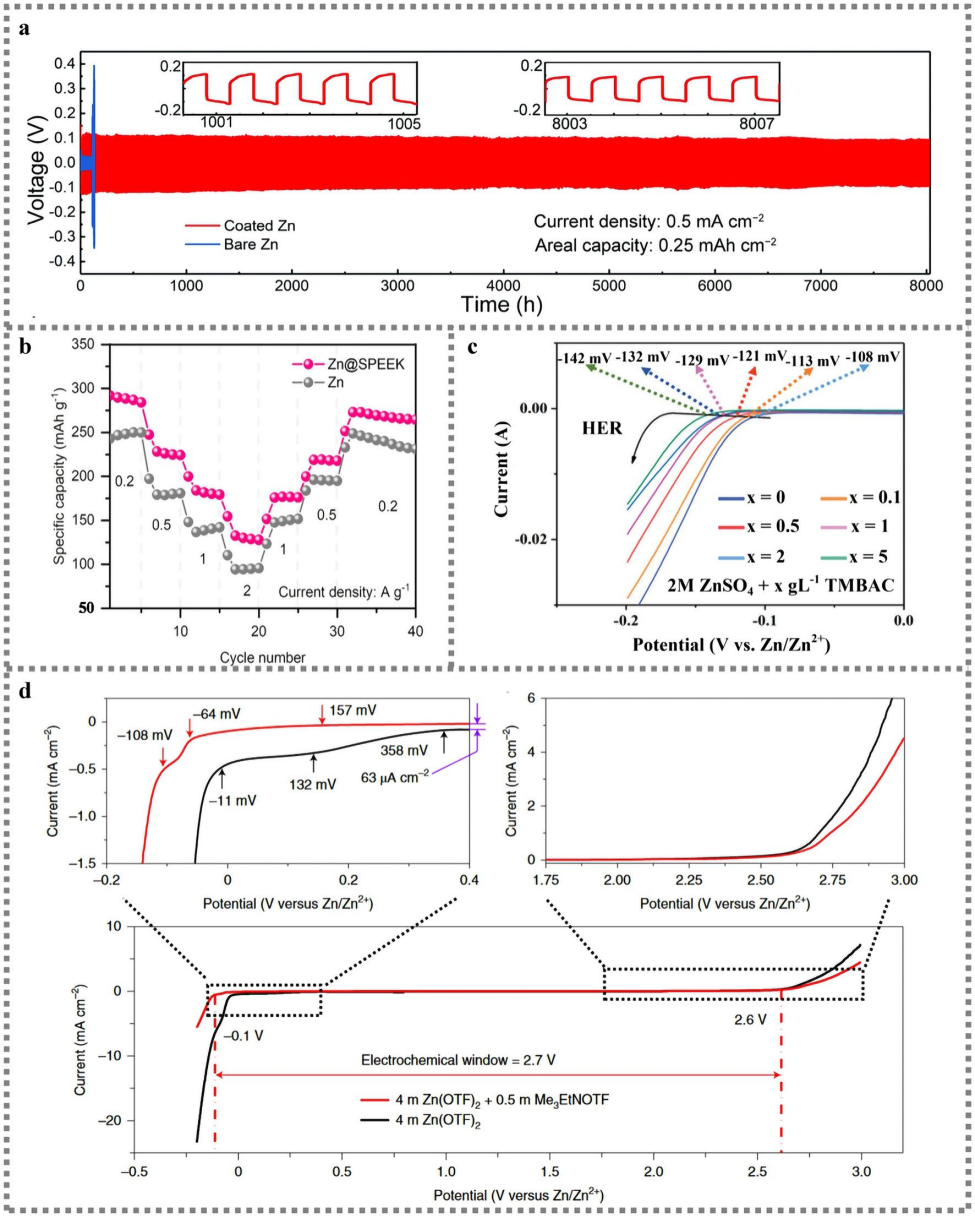
**a** Long-term galvanostatic cycling of symmetrical Zn cells with coated Zn plates and bare Zn plates [47]; Copyright 2019, Royal Society of Chemistry. **b** The rate performance of the full cells with varying Zn anodes operating [94]; Copyright 2021, ACS Publication. **c** LSV curves (HER) in ZnSO_4_ electrolyte with different TMBAC concentrations [96]; Copyright 2022, Wiley–VCH. **d** The electrochemical stability window of aqueous electrolytes measured using polarization scanning on non-active Ti electrodes versus Zn/Zn^2+^ [39]. Copyright 2021, Springer Nature

The cross-linking process is employed to combine the functionalized gelatin (Gel-MA) containing Zn salt with the Zn foil to form an artificial SEI film [91]. The symmetrical Zn||Zn battery containing artificial SEI film has a stable cycle over 2000 times. In sharp contrast, bare Zn has a short circuit after only 320 cycles. The developed artificial SEI film plays an important role in connecting Zn ions between the Zn anode and the electrolyte, which has the effect on isolating Zn anode from bulk aqueous electrolyte and preventing the Zn corrosion. Meanwhile, the multifunctional polymerized SEI layer interacts with Zn^2+^ through the functional catechol group on the polydopamine chain, which regulates the uniform Zn^2+^ distribution and promotes uniform nucleation [92]. Under high current density and high areal capacity of 30 mA cm^−2^, 30 mAh cm^−2^, it shows high plating/stripping reversibility over 1000 cycles exceeding 99.5% Coulombic efficiency, which fully demonstrates the feasibility of improved battery performance by SEI film. In situ formed Zn_3_(PO_4_)_2_ and ZnF_2_ (ZCS) are established on Zn anodes by using PF6^−^ anion induced chemical strategies to exploit the instability of KPF_6_ in the water environment. Stable plating/stripping over 2000 h is achieved in Zn||Zn symmetric cells with an average CE of over 99.37%. Meanwhile, the ZCS-Zn has strong reversibility and smooth & compact structure [93]. The Zn||Zn symmetric cell using Sac/ZnSO_4_ aqueous electrolyte achieves an ultra-long cycle life of 550 h at a current density of 10 mA cm^−2^ with a capacity of 10 mAh cm^−2^, which also achieves excellent stability over 220 cycles at the condition of 40 mA cm^−2^. Meanwhile, a high Coulombic efficiency of 99.6% is achieved using Zn||Cu half cells tested in the Sac/ZnSO_4_ electrolyte [40]. A polyamide coating layer elevating the nucleation barrier and restricting Zn^2+^ 2D diffusion, is constructed to effectively regulate the aqueous Zn deposition behavior [47]. The polymer-modified Zn anodes realize reversible dendrite free plating/spalling with cycle life over 8000 h (Fig. 9a). SEI plays an important role in zinc-based batteries. The presence of SEI improves the utilization of zinc during cycling and increases the depth of discharge of the battery when the battery is charged and discharged for a long period of time. Zinc is repeatedly deposited/dissolved when the battery is charged/discharged for a long period of time. The depth of discharge affects the cycle life of the battery. At high depth of discharge, SEI separates from the zinc electrode and zinc utilization decreases, leading to battery life decay. SEI is present to prevent side reactions during cycling, to protect the electrode, to improve zinc utilization and reversibility, and to maintain the steady state of the system.

### Rate Capability

An ultrathin (~ 1 μm) sulfonated poly(ether ether ketone) (SPEEK) SEI coated on the Zn anode surface can isolate water molecules and anions, and modulate Zn ion desolvation process [94]. As shown in Fig. 9b, the Zn||MnO_2_ full cells (127 mAh g^−1^ at 2 A g^−1^) has demonstrated an excellent rate capability. An artificial SEI layer of three-dimensional porous titanium material is developed to achieve high-rate capability with 95.46% capacity retained after 500 cycles at 5 A g^−1^ [95]. It originates from ultra-thin zincphilic titanium dioxide interface layer and continuous three-dimensional structure, which promotes the uniform Zn nucleation and homogenizes the current density. It is beneficial to the uniform Zn deposition and effectively restrain the dendrite growth.

### Electrochemical Stability Window

The electrochemical stability window is affected by both electrode and electrolyte. The main components of SEI films are determined by reduction products of solvents and additives in electrolytes, and SEI films in turn affect the electrochemical stability windows of electrolytes. The traditional aqueous Zn ion batteries system has the problem of narrow electrochemical stability window due to the water splitting. But various organic, inorganic solutions/salts, ionic liquid and long-chain polymers have been introduced to stimulate SEI formation or regulation solvation structure for wide electrochemical stability window [97]. Although organic electrolyte is conducive to in situ formation of SEI film, flammable factors also bring safety problems. The high-concentrated salt can provide a wide electrochemical window, but the high cost limits its large-scale application. SEI can effectively broaden the narrow electrochemical stability window of zinc battery, which isolates the direct contact between electrode and electrolyte, changes the original decomposition potential, avoids the decomposition of water and the corrosion of electrode, and improves the cycle life and capacity of the battery.

The benzyltrimethylammonium chloride (TMBAC, TMBA^+^ cation) as an additive is added to 2 M ZnSO_4_ solution to obtain a bright anhydrous Zn coating, which effectively inhibited the formation of dendritic and "dead Zn". The hydrophobic group of TMBA^+^ constructs a hydrophobic region in OHP, which hinders the decomposition of water molecules by regulating the potential of HER (Fig. 9c) [96]. Likewise, 0.5 M trimethylethyl ammonium trifluoromethanesulfonate as an additive is introduced to 4 M Zn(OTf)_2_ for in situ forming a hydrophobic fluoride interface, which extends the electrochemical stability window from 2.6 to 2.7 V (Fig. 9d) [39]. Although the SEI film has the ability to widen the electrochemical stability window of aqueous electrolyte, the scope of extension is limited.

SEI not only exists on the anode side, but also on the cathode side, SEI can broaden the electrochemical window [98]. In water system batteries, there is a problem of dissolution of cathode materials, which leads to the attenuation of battery capacity and greatly reduces the operating life of batteries. SEI on the cathode side prevents the active substance from being dissolved into the electrolyte, thus providing a protective layer and protecting the cathode material from being corroded by the electrolyte. The ideal cathode SEI, like the anode SEI, has low impedance, high ionic conductivity, prevents direct contact between the electrolyte and the electrode, good mechanical strength and flexibility, and prevents cracking when volume changes.

Qu et al. [99] use a polyimide (PI) precursor solution (poly((4,4′-carbonylbis(1,2-benzenedicarboxylic acid))-alt-(4,4′-methylenedianiline)) that allows for the fast formation (1 h) of the PI film. MnO_2_ nanowires are dispersed in the PI precursor solution and drawn into interlayer spaces in the micro-Swiss-roll current collector. After the rapid imidization of PI, MnO_2_ nanowires are coated onto the micro-Swiss-roll current collector. The nucleophilic oxygen in MnO_2_ attacks the C=O bonding to form O–C=O creating a zincophilic interface, a cation reservoir and a barrier to MnO_2_ dissolution. Guo et al. [100] report a cathode material of Ca_2_MnO_4_, they discover that the CaSO_4_·2H_2_O SEI film is generated on the surface of Ca_2_MnO_4_ during in situ charging process. The working mechanism is the intercalation of Zn^2+^ ions and Mn^2+^ ions, they can pass through SEI, but electrons cannot pass through it. At the same time, due to the presence of CaSO_4_·2H_2_O SEI, the capacity of the full battery does not decrease significantly after 1000 cycles at 1 A g^−1^.

As shown in Table 2, the working voltage of Zn batteries depends on both electrolyte and the cathode material. The electrolyte should provide sufficient electrochemical stability window for the redox reactions or insert/extract principle of cathode materials. The theoretical capacity is affected by the structure, component and electron transfer number of cathode materials. While the redox active site, reaction kinetics, electron transfer capability and loading mass directly affect the actual delivered capacity. Zn-based batteries have the problems of low output voltage and insufficient energy density, which limit their application in large-scale energy storage [113]. Many studies on high voltage and high capacity have been reported to solve these problems. A latent high-voltage MnO_2_ electrolysis process in a conventional Zn-ion battery is propose [114], the process of electrolysis of the Zn-MnO_2_ system is maximized by enabling proton and electron dynamics. By introducing Ni^2+^ into the electrolyte, the obtained Zn-Mn hybrid aqueous battery can be used to prove the catalytic kinetics of MnO_2_/Mn^2+^ electrolysis [115]. After the introduction of strongly electronegative Ni, the active electronic states and charge delocalization can be enhanced, the charge transfer of active O sites around the nickel dopant can enhance the catalytic electrolysis kinetics, and the electrochemical window was widened to 3.4 V.

**Table 2:** The performance of high-capacity, high-voltage aqueous Zn batteries [101]. Copyright 2022, Wiley–VCH

Cathode material	Battery configuration	Working voltage (V)	Capacity (mAh g^−1^)/energy density (Wh kg^−1^)	Mechanism	References
Co_0.247_V_2_O_5_·0.944H_2_O	Coin-type battery	1.15	432/497	Zn^2+^ intercalation	[102]
VO:CNT	Coin-type battery	1.21	487/589	Zn^2+^ intercalation	[103]
−MnO_2_	Coin-type battery	1.5	265/395	Al^3+^/Zn^2+^ intercalation	[104]
MnO_2_	Home-made battery (open system)	1.93	570/1100	MnO_2_/Mn^2+^ conversion	[104]
MnO_2_	Coin-type battery	1.41	430/602	MnO_2_/Mn^2+^ conversion and H^+^/Zn^2+^ intercalation	[105]
MnO_2_	Flow battery	1.58	616/973	MnO_2_/Mn^2+^ conversion	[106]
MnO_2_	Decoupled battery	2.71	609/1650	MnO_2_/Mn^2+^ conversion	[107]
MnO_2_	Decoupled battery	1.79	616/1100	MnO_2_/Mn^2+^ conversion	[108]
Graphite felt	Decoupled battery	2.53	–	Br_2_/Br^−^ conversion reaction	[108]
Carbon felt	Flow and decoupled battery	2.8	191/536	Ce_2_O^6+^/Ce^3+^ conversion reaction	[109]
Carbon felt	Flow and decoupled battery	1.76	359/632	Fe^2+^/Fe^3+^ conversion reaction	[110]
Porous carbon	Flow and decoupled battery	1.98	392/775	Br_3_^−^/Br^−^ conversion reaction	[111]
MnO_2_	Flow and decoupled battery	2.44	616/1503	MnO_2_/Mn^2+^ conversion	[112]

The diversified strategies from anode modification, electrolyte optimization, cathode material modification to new system design, is conducive to provides a new idea for the development of high-energy, high-power and long-lifespan Zn batteries.

## Design Strategies for SEI

SEI has an important influence on the performance of Zn -based batteries, but there are still some problems to be solved [116]. For example, the artificial SEI needs to be firmly bound to the Zn anode substrate, artificial SEI whether can exist in the electrolyte stability, mechanical properties of artificial SEI enough to prevent dendrite growth, whether the flexibility of artificial SEI can prevent cracking caused by the volume change, the ionic conductivity of artificial SEI is high enough to meet the migration of Zn ions, Whether the artificial SEI has stability in the long cycle of battery. The thickness of SEI formed *in* *situ* cannot be controlled, and the conditions required for the formation of SEI are harsh. For these problems, it is necessary to carry out a deeper study on the design of SEI, so that the theoretical design will gradually move to the road of practicality.

The ideal characteristics of SEI have been guiding the interface design. The anode and electrolyte are located on both sides of SEI film. So, the composition and structure of Zn anode and electrolyte will directly affect the properties of SEI film. For reported SEI interface design, artificial SEI membrane construction and in situ SEI membrane formation are the most common. The construction of artificial SEI membrane tends to modify and regulate the structure of Zn anode surface by an external protective layer. From the perspective of Zn ion and electron migration, it is beneficial to ion transport efficiency. Meanwhile, the SEI film will affect the ion distribution at the interface and nearby. In comparison, the in situ formation of SEI films is more dependent on the optimization of electrolytes, which subsequently affects the reductive and decomposed products with component or properties same to solvents and additives. By modifying the solvation structure, more free Zn ions are released for deposition and dissolution, thus improving energy utilization efficiency.

### Artificial SEI Film

For the structural design of the interface, the 3D porous structures are adopted to increase the transmission efficiency of Zn ions. The protective coating strategies successfully prevent the direct contact of Zn anode with electrolyte and partly hinder the occurrence of adverse events, which is beneficial to dendrite-free and hydrogen-free Zn uniform deposition. Although the appearance of artificial SEI membrane shows favorable factors for the improvement of battery performance, there are also some aspects required to be considered, e.g., the compatibility and matching problem between electrode and electrolyte, as well as the final device efficiency and cost, which requires more detailed research in the electrode structure design and interface modification. For instance, the ZnO@C core–shell nanorod is obtained by in situ growth of ZIFs on carbon cloth [117]. Then, three layers of 3D CC-ZnO@C-Zn anode is achieved after infusing Zn into it, which inhibits Zn dendrites growth and is conducive to the application in flexible energy storage system. The interface modification usually takes the form of anodic protective film. After depositing a uniform and highly viscoelastic polyvinyl butyral film on Zn surface, the artificial SEl film can isolate the Zn anode from water in the electrolyte [118]. Benefiting from its good adhesion, hydrophilic ion conductivity and mechanical strength, it induces the uniform stripping/electroplating of Zn ions. Similarly, the nano-barium titanate as SEI film (BTO@Zn) also can regulate the interfacial electric field and inhibit the growth of Zn dendrites [119]. Under the action of external electric field, BTO generates polarization and promotes evenly interfacial electric field distribution, which can guide the ordered transmission of Zn ions and improve the performance of Zn ion batteries. A hierarchical confinement strategy is proposed to design zincophilic and spatial traps through a host of porous Co-embedded carbon cages (denoted as CoCC) [120]. The zincophilic Co site has a low nucleation barrier and can be used as the preferred nucleation site, and the carbon cage can limit Zn deposition, the battery showed high rate performance and cycle performance.

### In Situ SEI Film

The formation of in situ SEI films is closely related to the decomposition of solvents and additives in electrolytes. The optimization of electrolytes is to change the solvation structure of Zn ions in the electrolyte, which can release more free Zn ions, broaden the electrochemical stability window of electrolyte, inhibit some side reactions and improve the reversibility of Zn anodes. But, there still is room for improvement of some properties such as ionic conductivity, thickness, and compactness. For example, the Zn_3_(PO_4_)_2_ with high interfacial energy could inhibit dendrite growth and the ZnF_2_ could accelerate the Zn^2+^ ions migration and suppress hydrogen evolution [93]. Constructing SEI films with Zn_3_(PO_4_)_2_ and ZnF_2_ as main components could comprehensively improve its kinetic properties. Using the instability of KPF_6_ in the water environment, ZnF_2_ is constructed in situ on Zn anode by the strategy of PF_6_^−^ anion induction. After several cycles, the reversibility of ZnF_2_-Zn is enhanced and the structure is compact and smooth. In addition, a dilute acidic Zn(OTf)_2_ aqueous electrolyte with Me_3_EtNOTF added can form a compact interface composed of ZnF_2_, ZnCO_3_, ZnSO_3_, and polyanions [39], enabling high reversible dendrite-free galvanization/stripping at high Coulombic efficiency near 99.9% CE, relatively broadening the electrochemical stability window and extending battery service life.

## Summary and Outlook

Zn ion battery is a new generation of energy storage device following Li ion battery, which not only performs well in energy density, but also shows well in safety and stability. An ideal stable SEI play critical roles in service life of battery and electrochemical stability window of electrolyte. It is necessary to explore the formation mechanism, micro conditions, electrolyte distribution and energy state of ions in electrolyte, and concrete forming process in detailed studies. In this article, we introduced the developing course of Zn batteries and SEI film formation mechanism firstly. Then, the in situ and ex situ characterization techniques are summed up to study the SEI film composition, microstructure, and morphology. We also briefly tell the story of low temperature hot electron microscope and other simulation research in the understanding of SEI. After that, we summarize the factors affecting the formation of SEI film from four aspects of anode materials, electrolyte, temperature and current density. As for the influence of SEI film on the performance of Zn ion battery, we summarized the recent related researches about improving the cycling performance of Zn anode, extending the electrochemical stability window, and prolonging the battery life. The ideal performance of SEI membrane also proposed for the design strategy of SEI membrane. The structural regulation and interface modification of Zn anode and electrolyte play critical roles to artificial SEI membrane and in situ SEI membrane construction.

Zn dendrite growth, HER and Zn corrosion reaction at the Zn/electrolyte interface are difficult problems to be solved to achieve high reversibility of Zn anode [121]. The decomposition voltage of water is 1.23 V, but it is usually higher than this voltage. Because of the existence of polarization, HER and OER exist in neutral, acidic or alkaline conditions. Therefore, the decomposition voltage of aqueous system includes the polarization of positive and negative poles and the polarization of electrolyte interface. Artificial SEI is a typical physical protection mechanism strategy. Many studies have confirmed that it can improve the dissolution efficiency of Zn deposition to a certain extent, has cyclic stability, and isolates the direct contact between electrode and electrolyte. However, there are still no mature design criteria for artificial SEI. The compatibility, chemical stability, high ionic conductivity, low electronic conductivity, compactness and mechanical flexibility of artificial SEI with electrodes and electrolytes are all factors to be considered in the design and manufacture. Compared with artificial SEI, in situ SEI formed by regulating solvation structure has good compatibility with electrolyte, but not all in situ SEI are solid and compact. Although a by-product layer Zn_4_SO_4_(OH)_6_·xH_2_O is formed at the interface between Zn and ZnSO_4_ electrolyte, the side reaction product is porous and microsized plate-like morphology, which cannot prevent the electrolyte from reaching the surface of Zn electrode, and the side reaction and hydrogen evolution reaction still occur. In addition, the formation process of SEI in situ is not a controllable process, so its structure, composition and thickness cannot be directly regulated. In addition, there is no fixed conclusion on the structure of in situ SEI at present, so it is necessary to carry out long-term research, so as to provide guidelines for the design of in situ SEI.

It also suggests that apart from the traditional method of Zn anode structure regulation and interface modification, and the optimization design of electrolyte, the design of SEI film can also do from the viewpoint of control of other factors, such as the regulation of temperature field, the electric field and magnetic field on the dynamics of Zn ions transportation and deposition/dissolution, and the stability of the system. The nucleation of Zn ion and its deposition at the anode are also worthy of our research. The internal relationship between the reduction and decomposition of different electrolytes should also be explored. More advanced characterization technology will provide greater convenience for our research and help us to have a more comprehensive understanding of SEI membrane.
